# Building a comprehensive cancer survivorship program

**DOI:** 10.3332/ecancer.2019.992

**Published:** 2019-12-12

**Authors:** Tessa Flores, Kathryn M Glaser, Douglas McDaniel, Denise Rokitka, Katharine A Amato, Mary E Reid

**Affiliations:** 1 Department of Medicine, Roswell Park Comprehensive Cancer Center, Buffalo, NY 14263, USA; 2 Department of Cancer Prevention and Populations Science, Roswell Park Comprehensive Cancer Center, Buffalo, NY 14263, USA; 3 Department of Pediatric Oncology, Roswell Park Comprehensive Cancer Center, Buffalo, NY 14263, USA; 4 Department of Health Behavior, Roswell Park Comprehensive Cancer Center, Buffalo, NY 14263, USA; 5 Department of Family Medicine, Primary Care Research Institute, University at Buffalo, Buffalo, NY 14260, USA

**Keywords:** cancer survivorship, integrative medicine, long-term survivors, quality of life, cancer continuum

## Abstract

There is a significant increase in the number of people surviving cancer as a result of improved detection and better treatments. In the United States (US) alone, these numbers are estimated to reach 20 million by 2026 [Miller *et al* (2016) *CA Cancer J Clin* 66(4) 271–289)]; [Bluethmann *et al* (2016) *Cancer Epidemiol Biomarkers Prev* 25(7) 1029–1036]. Living through cancer treatment represents a life-changing event, often including residual and long-term emotional, physical, psychological and spiritual sequelae. Survivorship programming must encompass the clinical management of medical issues, local support services for patients and their caregivers, protocols for communicating with community primary care providers (PCPs) and education for all clinicians in the survivorship continuum on the issues impacting survivors. This article will discuss a range of issues that should be addressed when developing a comprehensive, multi-disciplinary cancer survivorship care.

## Introduction: survivorship trends and cancer sequelae

The emphasis on developing survivorship programs is driven by the increasing numbers of cancer survivors. It is estimated that 20 million cancer survivors will be living in the United States (US) by the year 2026 [1, 2]. Patients are living longer, even with residual disease, with cancers that were previously considered terminal. For long-term survivors (LTS), there are several organisations with some general guidelines outlining survivorship care: the National Comprehensive Cancer Network (NCCN), the American Society of Clinical Oncology (ASCO) and the American Cancer Society (ACS). However, evidence-based guidelines are uncommon on how to best deliver these services for the detection of recurrent disease, management of symptoms resulting from treatment and support the emotional, physical, psychological and spiritual effects of cancer diagnoses and treatment. It is clear that this gap results in many unmet needs among LTS and their support systems [3, 4].

## Survivorship care models

Individualising and personalising survivorship care to your patient population is a critical first step in meeting the needs of the survivor population. Assessments on needs should be completed by all members across the survivorship care continuum, including patients and caregivers, to identify gaps in care and services. In addition, institutional and community providers should be included to determine educational opportunities and support services should be identified. Input from multiple shareholders, including oncology teams and community providers, is necessary to change patterns of care to accommodate survivor needs.

Several models of survivorship care programs exist which embed survivorship care within cancer centres, although few show how survivorship care can integrate into independent community practices. Free-standing or matrix cancer centres routinely have survivorship programs that are either: 1) decentralised where survivorship care happens in the primary oncology clinics; 2) centralised where all survivors come to one clinic coordinating survivorship care and 3) or a mixed model [5–8]. The structure is dependent on the size and location of the cancer centre, the preferences of the oncology teams and the potential numbers of survivors that will require surveillance care at the institution.

The program at Roswell Park Comprehensive Cancer Center (Roswell) is a mixed model of survivorship care. As a small, urban, free-standing comprehensive cancer centre, most solid tumour survivors, particularly breast, gynaecologic (GYN), head and neck (H&N), lung and some gastrointestinal (GI) cancers, are eventually transferred to the Survivorship Program for long-term follow-up care. This transitions patients from congested clinics whose primary focus is on newly diagnosed patients and those undergoing active treatment to a patient-centred care model focused on rehabilitation and wellness. As a comprehensive cancer centre, we have designed the program to meet national standards and guidelines, including NCCN, ASCO, ACS and the Commission on Cancer (CoC) (see Table 1). As the program is integrated into standard oncology practice, representing a culture change, it is necessary to have strong support and commitment from leadership.

However, all oncology teams do not incorporate survivorship care in the same way. Some disease sites prefer to monitor survivors in the original oncology clinic. At Roswell, this is the case for the haematologic cancers (leukaemia, lymphoma, multiple myeloma and bone marrow transplant) as well as genitourinary (GU) cancers. In contrast, the breast and gynaecology programs have embraced the transfer to survivorship as part of the long-term care pathway.

There are two ways for survivors to access the Survivorship Center (SC) at Roswell. The first starts as a one-time consultation at the beginning of the post-treatment period, where they can be immediately immersed in an array of support services. The second is a transfer of care to survivorship for continued surveillance of their primary cancer(s), to monitor for residual effects of treatment, to restart preventive cancer screening and to re-establish the connection with the primary care provider (PCP). Frequency of surveillance visits is based on NCCN guidelines for their cancer type, the tumour staging and response to treatments.

Other survivorship care delivery models focus long-term care and surveillance of survivors in community settings, including community oncology and primary care practices. If community oncologists are connected to a larger cancer centre network, such as a satellite or affiliate site, survivorship care can be incorporated within the network, moving towards patient-centred and personalised survivorship care. The alternative is for the PCP to take full responsibility for survivorship care, including surveillance and symptom management. This model allows oncology centres to decompress their patient load, easier access for patients and better monitoring of non-cancer comorbidities.

## Sharing care of patients

A cornerstone to quality survivorship care is establishing a strong relationship between the PCP, the oncology team, the survivorship team and the patient. When patients are initially diagnosed with cancer and are referred to oncologists, the PCP often plays a secondary role in patient care, more frequently than with other medical specialities where more of the care is shared with the PCP [9]. When cancer treatment is finished, re-engaging the PCP requires targeted communication. Transitioning survivorship care to the PCP can be complicated because PCPs do not necessarily incorporate the survivorship guidelines into a standard of care. In addition, there is little guidance from experts, like oncologists, and most electronic health record systems do not support cancer-specific guidelines [9].

The survivorship care plan (SCP) is a means of communication that promotes a shared-care coordination model [9, 10]. As research on SCPs has shown, the plan alone does not relieve stress for survivors [19, 20]. However, it provides information to the PCP on treatment received, potential long-term sequelae, and provides a road map of survivorship care that improves their level of confidence in managing survivors. Although early research on SCPs was not done in the context of offering quality survivorship care, the document itself may positively impact patients if they receive the services and support recommended in the SCP. At Roswell, our providers have generated 3,600 care plans and effectively use the SCP to communicate follow-up plans with their patients. The SCP standard should highlight the cancer surveillance schedule, support service referrals, cancer screening recommendations, potential long-term complications related to the treatment and recommendations regarding monitoring of long-term complications to the PCP [11].

In addition, the SCP should also specify what testing should be done and who should be doing it, to avoid duplication of effort. For example, for breast cancer survivors on long-term hormonal therapy, lipid profiles should be done by the PCP since they are considering other cardiovascular risks and conditions not related to cancer. At Roswell, our patient navigator ensures that the correct PCP is recorded in the electronic medical record, which in turn guarantees that the correct PCP receives correspondence and the personalised SCP for each LTS. The patient navigator also facilitates a new patient appointment with a PCP if needed to ensure continuity of care.

However, there is a growing literature supporting that PCPs are not always prepared to address the needs of cancer survivors [8, 12–15]. There is also evidence that the relationship between patients and their PCPs is neglected during cancer treatment and, thus, the PCP is not always aware of the status of their patients as they complete treatment. The Institute of Medicine’s 2006 seminal report, which continues to be relevant today, highlighted how the transition of care from oncology to primary care is often fragmented and lacks coordination [8, 12].

PCPs often do not have a complete treatment summary from oncologists, which also limits their ability to monitor side effects from treatment. Table 2 summarises the most common side effects of cancer treatment. As a result, many cancer survivors feel uncertain about their care once they are in transition from oncologists to PCPs [16]. In addition, PCPs may not be up to date on the latest treatments for side effects or the current trends in surveillance and survivorship guidelines. It is essential that the oncology or survivorship clinical teams communicate clearly with the PCPs on the surveillance plans for survivors re-entering their PCP care. In addition, educational programs on survivorship can provide PCPs with the skills to follow surveillance guidelines as well as to detect and identify any new cancers.

## The structure of survivorship care

There are many options for structuring survivorship care. Based on our experience in developing a new program, we considered several goals of care. It is important to provide patients and their caregivers with patient-oriented care. Each visit should include a comprehensive physical and psychological assessment. Access must be provided to support services for both patients and caregivers. It is critical to individualise survivorship care with the use of care plans (SCP) that provide a roadmap for future care and services to facilitate communication between the oncology teams and community-based PCPs. It is also important to maximise the level of health and wellness for every survivor and their support network through education, wellness activities and integrative medicine.

Roswell’s SC opened in January 2017 for any patient who would benefit from survivorship and supportive care services. Clinical assessments are supplemented with appropriate support services utilising specialised resources (nutrition, rehabilitation, acupuncture, social work, psychology and wellness programs) in order to improve the quality of life (QoL) for survivors, caregivers and their support networks. This includes actively coordinating surveillance and follow-up care with PCPs and other community providers. The initial target population was primarily cancer survivors who had completed treatment and were outside a surveillance window that could be permanently transferred to the SC for long-term follow-up care. Now patients can be referred to survivorship upon completion of treatment to receive an SCP and be introduced to the survivorship care model, start rehabilitation and wellness, and return to the oncology clinic for follow-up until the oncologist transfers them to the SC for long-term survivorship care.

The survivorship clinical team consists of a medical director who serves as an oncogeneralist (board-certified in internal medicine and paediatrics) for the adult patients, a paediatric oncologist for the paediatric survivors under 40 years and young adult survivors (under 40 years at the time of diagnosis), a family practitioner for paediatric survivors over the age of 40, an oncology trained nurse practitioner (NP), clinical nurse care coordinators and a patient care navigator. Scheduling templates were adjusted according to volumes to make sure that we were not overstaffing the clinic. As of July 2019, 2,436, survivors have been seen in the clinic. The majority of these cancer survivors were breast (54%), paediatric (20%), GYN (17%), as well as adolescent and young adult (3%) cancer survivors. Other cancer disease sites (6%) that have transferred care for the LTS are gastroenterology (GI), thoracic, GU, and head and neck (H&N) cancers.

Our survivorship team also includes a nutritionist, social workers, psychologists and integrative medicine providers. The team monitors for recurrence of cancer and new cancers, reviews the recent medical history and completes a thorough physical examination. In addition, the team identifies and manages the side effects of cancer, providing counselling on healthy living habits, such as diet and exercise, and referrals to screening tests for other cancers, and connects patients with PCPs and/or outside experts.

## Assessment tools: the quality of life questionnaire

The European Organisation for Research and Treatment of Cancer (EORTC) Quality of Life Questionnaire – Core 30 (QLQ) is a widely used QoL questionnaire in cancer care [15, 17]. At every routine surveillance visit, the cancer survivor completes this questionnaire prior to seeing the provider. The EORTC QLQ assesses five domains including physical function, emotional function, role function, fatigue, pain and global function. These domains are summarised, and clinical thresholds prompt further inquiry and possible referrals based on four domains (physical function, emotional function, fatigue and pain) [7].

Depending on their EORTC QLQ scores, LTS are assessed and referred to physical therapy, occupational therapy, bioenergy therapies (acupuncture, Reiki) and psychosocial counselling. As of December 2018, 1,160 LTS completed the EORTC QLQ at least once and, when combined with tumour registry information, allows us to explore trends from the QoL assessments to better understand the needs of our patient population and adjust the services available to them.

## Identifying support services

Whether survivorship care happens within an institution or in the community, it is critical to identify an array of support services that can address the needs of survivors in advance of opening the program. At Roswell, most of these services were already available but not organised around survivorship care. Coordinating these various services required several meetings to educate the team on their role in care and to work out the pathways for referrals. In a community-based care setting, this process is comparable. Wherever the services are based, there is some vetting and training required to make sure that the various services understand the impact of cancer and the specific needs of cancer survivors. For example, physical therapists must be able to set appropriate performance and training standards to accommodate physical limitation from cancer surgeries or other therapies.

In November 2015, Roswell’s Marketing and Communications Department mailed 35,420 letters to all current living patients and invited them to participate in an online survey to help develop the survivorship program. Patients were informed that the purpose of the survey was to gather information on interest in specific programs and their response would influence the services offered to Roswell Park survivors through the survivorship program. A total of 1,054 surveys were completed, resulting in a 3% response rate. Table 3 summarises the 10 most frequently requested support services by survey 3 respondents. This survey laid the foundation of the support services offered to LTS through the program.

The clinical space that houses both the Survivorship and Palliative Medicine Programs is a central hub for these support services. Psychiatry, psychology, social work, nutrition, acupuncture and biofield therapies are physically housed in the centre. Other support and wellness programs, such as physical rehabilitation, yoga, massage, meditation, support groups and education groups, are offered throughout the cancer centre. See the summary of the available support services in Table 4. All these support services are offered to LTS and caregivers. To address the spiritual concerns of our LTS, we refer them to our pastoral care program within the cancer centre which includes a chapel open to our patients and families and. This is a multi-denominational program that services our inpatient units, but appointments can be made for our LTS during their clinical visits. This service has provided special activities for survivors, such as Yoga and storytelling, which are aimed at providing a venue to discuss spiritual issues. To expand specialised programs to meet the ongoing needs of LTS, Roswell is now developing a sexual health clinic. This clinic will have GU and GYN-trained surgeons to address sexual dysfunction issues and an NP and psychologist to provide sexual health therapy and support for LTS.

## Screening for other primary cancers

In addition to continued surveillance of their original cancer as dictated by NCCN guidelines, LTS need standard cancer screening. About 80% of newly diagnosed cancers are found in individuals with a prior history of cancer [18]. This makes surveillance for a second primary cancer a requirement for all survivorship programs. Individual patient needs for breast, colon, cervical, oral, prostate and lung cancer should be evaluated. The survivorship program shares a staff and physical space with the cancer screening program, resulting in a seamless process for facilitating guideline-driven cancer screening. In a community setting, the PCP should be aware of current screening guidelines and be encouraged to review adherence annually.

Importantly, cancer screening is scheduled at the convenience of the patient. We have partnered with two community gastroenterology practices to provide screening colonoscopy. Our patients go directly to the colonoscopy procedure at a location, streamlining the process and making it more convenient. We can also arrange mammograms on the same day as the survivorship appointment. If LTS meet the criteria for lung cancer screening, we order the first initial low-dose CT and then navigate them to the High Risk Lung Cancer Screening Clinic at Roswell for further evaluation and management. We also perform Pap tests for cervical cancer screening and digital rectal exams for early prostate detection, and then navigate patients back to community providers for further follow-up. Finally, the genetic testing service is embedded in the cancer screening program. This facilitates genetic testing, including the rescreening of patients who had BRCA1/2 prior to 2012 according to new standards and guidelines.

## Refining survivorship care

As standards for survivorship care are refined and expanded throughout the US, there are many lessons that can be learned from the innovative approaches developed in England and Ireland [3, 4]. The published proceedings from ASCO and the ACS described the collaborative efforts led by the National Cancer Survivorship Initiative (NCSI) and National Health Service (NHS) to initiate change in post-treatment recovery care and the risk stratification of survivors [4]. The program to develop personalised care pathways rests on four basic elements: 1) a comprehensive assessment of the survivor; 2) generating and sharing a treatment summary with patients and community-based PCPs; 3) reviewing the summary with the patient and 4) supporting community events to inform survivors of coping strategies and promoting wellness. In conjunction with this strategy, the NCSI stratified cancer survivors into three risk categories (low, moderate and high) based on their cancer, their treatment and response and the degree of exposure to toxicity causing regimens. As outlined by Alfano *et al* [4], the intensity of surveillance of survivors by risk level is both a sound concept and more cost-effective.

There are several sequelae that can impact LTS QoL over time and require vigilant surveillance and coordination of multiple services. Cardiotoxicities, for example, can appear decades after the exposure to cardiotoxic therapies. Many cancer treatment therapies, with the exception of many surgeries, can impact post-treatment fertility. Symptoms such as xerostomia (dry mouth resulting from reduced or absent saliva flow) can occur in head and neck survivors 5–10 years post-treatment. It is important that survivorship programs are aware of the potential long-term effects of cancer treatment and develop collaborations with specialists prepared to address these issues. Oncocardiologists, endocrinologists, dentists and fertility specialists are examples of the more common specialists who can contribute to providing coordinated survivorship care. These providers can work hand-in-hand with the survivorship team and PCPs to provide comprehensive care. It is imperative that all members of the survivorship care team, regardless of where they practice, be aware of the early signs of these sequelae.

## The role of survivorship research

Clinical research is the key to our ability to close the gaps in understanding the best way to treat survivors and improve survivorship care. Clinical programs must be structured to seamlessly incorporate clinical research. It is critical that an evidence base can be developed to guide the development of effective health care models, risk models of survivor co-morbidities and mortality, optimal times to intervene with the best treatments, to the longest overall survival and highest QoL for survivors. Finally, research must clarify the capacity of LTS to change their behaviours, both from a disease prevention and a health promotion standpoint [11]. While survivorship programs embedded in cancer centres can offer direct access to a large population of survivors, survivorship research must also be embedded in community settings.

## Summary

With the increasing number of people surviving cancer, it is important to plan for the long-term care and symptom management needs of this population. In this decade, survivorship care development must 1) account for the acuity of survivors; 2) identify the levels of risks of persistent or severe toxicities; 3) designate who will provide survivorship care, including the management of unique medical and psychological issues resulting from cancer treatment; and 4) determine the availability of the supportive services needed by survivors and their caregivers. Ultimately, research must clarify both the best health care models for caring for survivors and the optimal regimes that will result in the highest QoL for this growing population.

## Conflicts of interest

There are no conflicts of interest to declare for any of these authors relative to this work.

## Funding

This work was supported by Roswell Park Comprehensive Cancer Center and NCI grant P30CS016056. The Integrative Medicine program is supported by the Roswell Park Alliance Foundation.

## Authors’ contributions

Tessa Flores, Kathryn M Glaser and Mary E Reid contributed equally to the writing of this paper. Douglas McDaniel, Denise Rokitka, and Katharine A Amato reviewed and contributed to the editing of the text.

## Figures and Tables

**Table 1.** Examples of websites of survivorship treatment guidelines and comprehensive programs.

**ASCO Survivorship Compendium**	https://www.asco.org/practice-guidelines/cancer-care-initiatives/prevention-survivorship/survivorship/survivorship-compendium	The Survivorship Care Compendium has been developed to serve as a repository of tools and resources to enable oncology providers to implement or improve survivorship care within their practices. The compendium serves as an accompaniment to the educational opportunities and clinical-guidance ASCO offers on survivorship care. Although ASCO endorses the National Coalition for Cancer Survivorship definition of a cancer survivor as starting at the point of diagnosis, the focus of this compendium is on individuals who have completed curative treatment or who have transitioned to maintenance or prophylactic therapy.
**Survivorship, version 2018, NCCN clinical practice guidelines in oncology**	https://jnccn.org/view/journals/jnccn/16/10/article-p1216.xml	The NCCN guidelines for survivorship provide screening, evaluation and treatment recommendations for common physical and psychosocial consequences of cancer and cancer treatment to help healthcare professionals who work with survivors of adult-onset cancer in the post-treatment period. This portion of the guidelines describes recommendations regarding the management of anthracycline-induced cardiotoxicity and lymphedema. In addition, recommendations regarding immunisations and the prevention of infections in cancer survivors are included.
**Patient and survivor care**	https://www.asco.org/practice-guidelines/quality-guidelines/guidelines/patient-and-survivor-care	Clinical practice guidelines serve as a guide for doctors and outline appropriate methods of treatment and care. Guidelines can address specific clinical situations (disease-oriented) or use of approved medical products, procedures or tests (modality-oriented). Multidisciplinary panels of experts, including patient advocates, develop ASCO’s clinical practice guidelines.
**Mayo clinic survivorship program**	https://www.mayoclinic.org/departments-centres/oncology/cancer-survivorship-clinics/overview	Mayo Clinic, based in Minnesota, US has a well-established survivorship program, which is presented on their easy to navigate website. Survivorship care is distributed in disease-specific clinics.
**Memorial Sloan Kettering Cancer Center (MSKCC) survivorship program**	https://www.mskcc.org/experience/living-beyond-cancer/survivorship	MSKCC survivorship and follow-up care programs can help cancer survivors lead a healthy, active life after cancer as well as manage any problems related to cancer therapy. The Adult Survivorship Program offers comprehensive services to people who have completed cancer treatment. The program focuses not only on cancer screening but also on overall health and wellness. Most survivors in our program were diagnosed as adults. There are also comprehensive programs for patients diagnosed as young adults and children.
**Prevention and monitoring of cardiac dysfunction in survivors of adult cancers**	https://www.asco.org/practice-guidelines/quality-guidelines/guidelines/patient-and-survivor-care#/14726	Cardiac dysfunction is a serious adverse effect of certain cancer-directed therapies that can interfere with the efficacy of treatment, decrease quality of life or impact the actual survival of the patient with cancer. The purpose of this effort was to develop recommendations for prevention and monitoring of cardiac dysfunction in survivors of adult-onset cancers.
**Head and neck cancer survivorship care**	https://www.asco.org/practice-guidelines/quality-guidelines/guidelines/patient-and-survivor-care#/24416	This guideline provides recommendations on the management of adults after head and neck cancer (HNC) treatment, focusing on surveillance and screening for recurrence or second primary cancers, assessment and management of long-term and late effects, health promotion, care coordination and practice implications.
**London cancer alliance**	http://www.londoncanceralliance.nhs.uk/information-for-patients-public/information-support/survivorship/	Provides guidelines and practical information on issues related to survivors.
**Nation Institute for Health Care Excellence**	https://www.evidence.nhs.uk/search?q=cancer+survivorship	UK-based organisation that provides published evidence supporting survivorship care.
**American Cancer Society Guidelines for Survivorship**	https://www.cancer.org/health-care-professionals/american-cancer-society-survivorship-guidelines.html	The cancer survivorship care guidelines address surveillance for recurrence, screening for second primary cancers, assessment and management of physical and psychosocial long-term and late effects of cancer and its treatment, health promotion, care coordination and practice implications.
**Roswell Park Comprehensive Cancer Center Survivorship Program**	https://www.roswellpark.org/survivorship	Roswell Park provides a comprehensive survivorship program including clinical and support services.

**Table 2.** List of treatment sequelae for cancer survivors.

Anxiety	Fatigue	Occupational problems
Body Image distortion	Fertility issues	Pain
Cardiotoxicity	Financial toxicity	Post-traumatic stress disorder
Dental problems	Legal concerns	Second primary tumours
Depression and anxiety	Lymphedema	Sexual function issues
Diarrhoea/incontinence	Neuropathy	Sexual intimacy issue
Family problems	Nutritional problems	Weight gain

**Table 3.** The top ten support services requested by cancer survivors.

Service	Percentage (%)
Nutrition information	39.1%
Yoga for physical fitness	30.9%
Cooking classes	26.4%
Aerobics	26.2%
Meditation	25.9%
Yoga for stress reduction	25.3%
Swedish massage	24.5%
Nutrition consultations	24.5%
Special diets information	24.1%
Information on herbal and dietary supplements	23.6%

**Table 4.** Array of support services available to cancer survivors.

Acupuncture	Mindfulness	Reiki
Cancer screening	Nutrition	Smoking cessation
Fertility counselling and preservation	Occupational therapy	Social work
Genetic testing and counselling	Physical therapy/rehabilitation	Support groups
Massage	Psychiatry	Yoga
Meditation	Psychology	Young adult (YA) specialty
